# Alterations of the paired maternal vaginal microbiome and neonatal meconium microbiome in vulvovaginal candidiasis positive pregnant women

**DOI:** 10.3389/fcimb.2024.1480200

**Published:** 2024-12-13

**Authors:** Hongqin Zhang, Hongping Li, Ruolin Zhang, Lingxia Ji, Jun Chen, Chuan Nie, Weimin Huang

**Affiliations:** ^1^ Neonatology Department, Shenzhen Nanshan Maternity and Child Healthcare Hospital, Shenzhen, China; ^2^ Neonatology Department, Affiliated Shenzhen Children's Hospital of Shantou University Medical College, Shenzhen, China; ^3^ Neonatology Department, Guangdong Women and Children Hospital, Guangzhou, China

**Keywords:** vulvovaginal candidiasis, vaginal microbiome, meconium microbiome, microbial community, genital infection

## Abstract

**Background:**

Women with vulvovaginal candidiasis (VVC) are known to experience vaginal microbial dysbiosis. However, the dynamic alterations of the vaginal microbiome in pregnant women with VVC and its effect on neonatal gut microbiome remain unclear. This study aims to characterize the vaginal microbiome in pregnant women with VVC and its impact on their offspring’s meconium microbiome.

**Methods:**

Forty-four pregnant women, including 17 with VVC (VVC group) and 27 healthy controls (HC group), along with their 44 offspring, were enrolled in this study. Maternal vaginal samples were collected during the pre- and post-delivery phases. Meconium samples from their newborns were also obtained. Microbial communities were characterized using 16S rRNA sequencing.

**Results:**

The vaginal microbiome of healthy pregnant women was predominantly composed of the genus *Lactobacillus*. The Bray-Curtis dissimilarity index indicated significant alterations in the vaginal microbiome of the VVC group, with a notable decrease in *Lactobacillus* and significant increases in *Delftia*, *Burkholderia* during both the pre- and post-delivery phases compared to the HC group. Additionally, the neonatal meconium microbiome exhibited significant differences between the VVC and HC groups, with *L. salivarius* and *L. helveticus* significantly decreased and *Delftia* significantly increased in the VVC group. Similar trends in microbial variation were observed across maternal and neonatal microbiomes, indicating intergenerational concordance associated with VVC.

**Conclusion:**

VVC alters the microbiota of both pregnant women and their neonates at birth, suggesting a form of microbial inheritance. These findings underscore the distinctive characteristics of the vaginal microbiome associated with VVC and its potential impact on the formation of early-life gut microbiome.

## Introduction

The vaginal microbiome during pregnancy is of considerable interest due to its impact on both maternal and neonatal health (Fettweis et al., 2019; Zhao et al., 2021). The maternal vaginal microbiome may serve as an important source of pioneer bacteria for the neonatal gut microbiome (Dominguez-Bello et al., 2010; Kervinen et al., 2019), profoundly affecting host metabolism and immunity (Gaziano et al., 2023) and improving neonatal neurodevelopment (Zhou et al., 2023). During a healthy pregnancy, the vaginal microbiome is characterized by low bacterial species diversity and is typically dominated by one of several different species of *Lactobacillus* (Ravel et al., 2011; Zhang et al., 2022a). Researchers have categorized the vaginal microbiome into five community state types (CSTs) based on the dominant bacterial species. CST I, CST II, CST III, and CST V are dominated by *L. crispatus*, *L. gasseri*, *L. iners*, and *L. jensenii*, respectively. In contrast, CST IV is characterized by the absence of *L.* spp. and comprises a diverse array of strict and facultative anaerobes (Ravel et al., 2011; DiGiulio et al., 2015; MacIntyre et al., 2015). The composition of the vaginal microbiome is influenced by genetic factors, such as ethnicity, as well as environmental, individual and lifestyle factors (Kervinen et al., 2019). Additionally, infectious diseases and their treatments play a significant role in shaping the vaginal microbiome (Greenbaum et al., 2019; Chen et al., 2021b; Zhang et al., 2022b).

Vulvovaginal candidiasis (VVC) is a multifactor infectious disease affecting the lower reproductive tract in women, predominantly caused by *Candida albicans*, and results in pathological inflammation (Farr et al., 2021). VVC is one of the most common forms of infectious vaginitis, with an estimated 75% of women experiencing at least one episode in their lifetime. Recurrent VVC affects nearly 8% of women worldwide (Denning et al., 2018). Vulvovaginal yeast infections caused by *Candida* species are more prevalent in pregnant women compared to non-pregnant women, potentially due to hormonal and immunological changes that occur during pregnancy (Aguin and Sobel, 2015; Chatzivasileiou and Vyzantiadis, 2019). These infections during pregnancy are common and can lead to extensive inflammation, which may contribute to adverse perinatal outcomes (Chatzivasileiou and Vyzantiadis, 2019; Schuster et al., 2020).

Accumulating studies have demonstrated alterations in vaginal microbial communities during genital infections, including VVC, bacterial vaginosis infection, and *Chlamydia trachomatis* infection (Ceccarani et al., 2019; Zhao et al., 2023). These microbial communities are also affected by drug treatments (Xiao et al., 2022). Moreover, the vaginal microbiome is dynamic during and after pregnancy and in the postpartum period (MacIntyre et al., 2015; Li et al., 2022). However, information regarding dynamic changes in maternal vaginal microbiome due to VVC is limited, and its effect on the gut microbiome of offspring remains to be understood. Therefore, this study aimed to compare the alterations in maternal vaginal microbial composition before and right after delivery in pregnant women with VVC versus those without VVC. Additionally, the meconium microbiome was also compared between the offspring of VVC and those without.

## Methods

### Study subjects

Pregnant women and neonates were recruited at Shenzhen Nanshan Maternity and Child Health Care Hospital. The study protocols were approved by the ethics committee of Shenzhen Nanshan Maternity and Child Health Care Hospital (No. NSFYEC-KY-2022018) and conducted in accordance with all relevant guidelines and regulations. Written informed consent was obtained from all participants, and for neonates, consent was provided by their parents or legal guardians. Participants who did not consent to participation were excluded from the study. Additionally, participants were excluded if they had taken antibiotics or amicrobial therapy during pregnancy, consumed probiotics within three months before delivery, experienced adverse pregnancy outcomes, had a history of smoking and alcohol consumption during pregnancy, had complicated singleton pregnancies, had any underlying medical diseases prior to pregnancy, or if the newborn had physical abnormalities.

A total of forty-four healthy women with a gestational age (GA) greater than 37 weeks who delivered vaginally at maternity centers were included. Specifically, 17 pregnant women were classified into the VVC group, while the remaining 27 pregnant women were designated as the normal controls (HC group). VVC diagnosis was established through microscopic and culture-based identification of *Candida*, as previously reported (Ceccarani et al., 2019). All pregnant women were tested at 35-36 weeks of gestation at our hospital, and only asymptomatic, without therapeutic intervention VVC cases recruited to minimize the interference of external factors on study outcomes.

### Sample collection

Vaginal swab samples were collected from all study participants in two different phases: upon admission to the hospital for delivery (phase BD, before delivery), and immediately after delivery (phase PD, after delivery). To ensure sufficient DNA concentration, three sterile cotton swab sticks were used to collect vaginal swab samples by trained nurses. Following a previously reported method (Li et al., 2023), the sterile swab was placed carefully on the vaginal sidewall about halfway between the introitus and the cervix, and rolled dorsally-ventrally back and forth four times to coat the swab.

Meconium samples were obtained within 24 hours after delivery and collected in a sterile tube under antiseptic handling by trained nurses according to previously reported (Turunen et al., 2021). All samples were stored at -80°C until DNA extraction.

### Microbiome profiling

Microbial DNA was extracted using the QIAamp DNA Mini kit, and its concentration and purity were measured using the NanoDrop One (Thermo Fisher Scientific, MA, USA). The V3-4 variable region of the 16S rRNA gene was amplified using forward primer 338F (5’-ACTCCTACGGGAGGCAGCAG-3’), and the reverse primer 806R (5’-GGACTACHVGGGTWTCTAAT-3’) (Chen et al., 2021a). PCR reactions, containing 25 μL 2× Premix Taq (Takara Biotechnology, Dalian Co. Ltd., China), 2 μL of each 10 mM primer, and 3 μL DNA template in a volume of 50 μL, were amplified under the following thermal profile: 94 °C for 5 minutes, then 30 cycles of 94 °C for 30 seconds, 52 °C for 30 seconds, 72 °C for 30 seconds, followed by 72 °C for 10 minutes. PCR products were then pooled in equimolar and paired-end sequenced on an Illumina MiSeq platform with V3 chemistry.

The 16S rRNA gene sequences were analyzed using the bioinformatics software package QIIME2 (version 2021.8) (Bolyen et al., 2019). Paired-end reads were denoised by QIIME2 using the command “qiime dada2 denoise-paired”, which aimed to merge paired-end reads, perform quality filtering, and exclude chimeric and phiX sequences. Taxonomic assignment was performed against the Greengenes (13_8 revision) database using command “qiime feature-classifier classify-sklearn”. Alpha- and beta-diversity measures were generated using the commands “qiime phylogeny align-to-tree-mafft-fasttree” and “qiime diversity core-metrics-phylogenetic”.

### Statistical analysis

Continuous variables were presented as the mean ± standard deviation (SD), and categorical characteristics were reported as numbers (percentages). All comparisons were performed using R software at a significant level of 0.05, with chi-square and t-tests for categorical and continuous variables, respectively.

Principal coordinate ordination analysis (PCoA) was performed on Bray-Curtis distances and accompanied by permutational multivariate analysis of variance (PERMANOVA, 999 permutations) using the R package “vegan” and MicrobiotaProcess (Xu et al., 2023). Differentially abundant taxa between the two groups were estimated using LEfSe (Linear Discriminate Analysis of Effect size) software, with a significance threshold of P < 0.05 and LDA > 2.0 (Segata et al., 2011).

## Results

### Demographic and clinical data of study subjects

**Figure 1:**
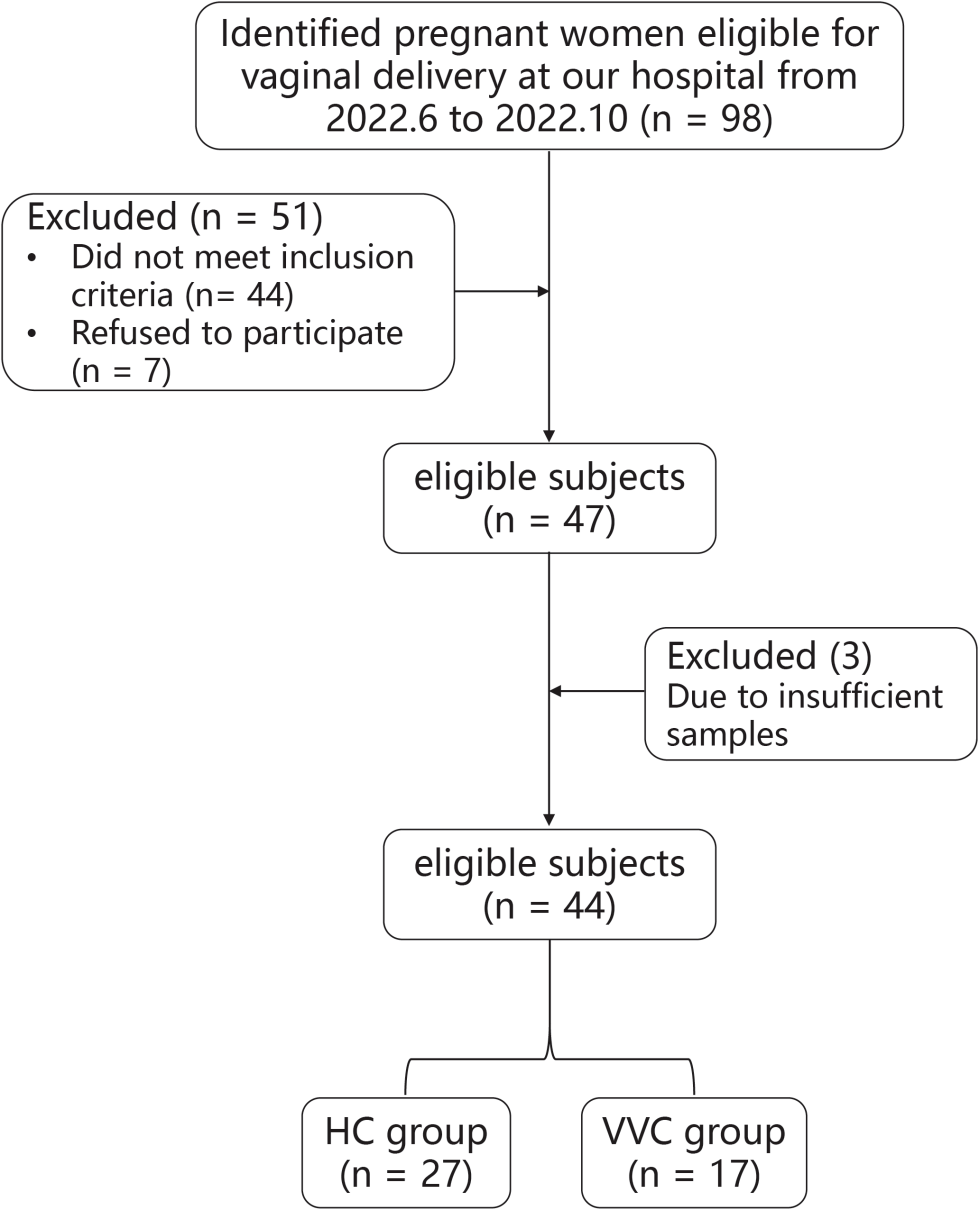
The flow scheme of the study population.

The flowchart illustrating the subject recruitment and exclusion process was detailed in **Figure 1**. Ultimately, forty-four pregnant women (VVG group, n = 17; HC group, n = 27) were included in this study. All participants were Han Chinese residing in Shenzhen city. The characteristics of all participants were summarized in **Table 1**, there were no differences in terms of maternal age, gestational age, neonatal birth length and neonatal gender. Due to insufficient DNA in some meconium samples, only 16 neonates in the VVC group and 17 neonates in the HC groups were retained for further analysis. Then in the data analysis process, two steps were undertaken: first, the number of maternal vaginal samples was adjusted to match the number of newborn meconium samples, and second, all maternal vaginal samples were standardized to ensure consistency across groups.

**Table 1:** Demographic and clinical data for study participants.

Characteristics	VVC group (N = 17)	HC group (N = 27)	P value
Mother’s age, years mean (SD)	31.06 ± 3.68	31.5 ± 3.65	0.73
Pregnancy weight gain, kg mean (SD)	12.0 ± 4.57	14.9 ± 5.32	0.12
	VVC group (N =16)	HC group (N = 17)	P value
Neonatal’s birth weight, g mean (SD)	3160.0 ± 203.5	3261.9 ± 326.5	0.52
Neonatal’s gender (male/female)	5/11	8/9	0.36
Gestational age, weeks, (mean, SD)	39.54 ± 0.98	39.78 ± 0.86	0.48
Neonatal’ weight postpartum 6 months, g mean (SD)	8128.6 ± 749.8	8701.5 ± 779.6	0.06

### Comparisons of vaginal microbiome before delivery between the VVC and HC groups

**Figure 2:**
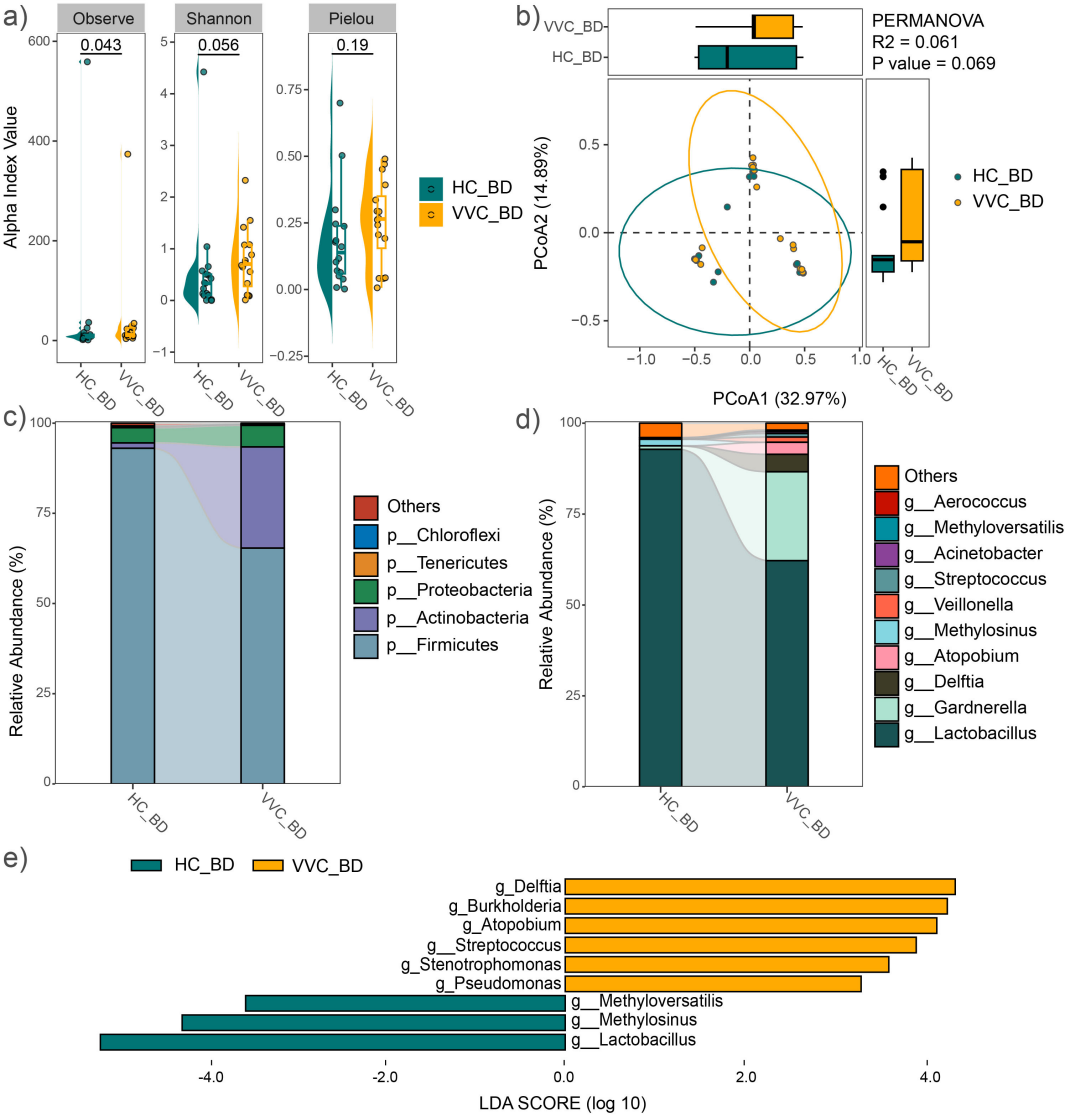
Comparison of vaginal microbial community before delivery between the VVC and HC groups. **(A)** Comparisons of alpha diversity indices. **(B)** PCoA plot illustrating the differences in microbial communities between the two groups. **(C)** Relative abundances of the dominant phyla. **(D)** Relative abundances of the predominant genus. **(E)** Significant different genus between the two groups.

Alpha diversity was assessed using the observed feature number, Pielou index, and Shannon index. The results demonstrated that the mean values of the observed feature number significantly increased in the VVC group compared to the HC group (P = 0.043), while no significant differences were observed in the Pielou (P = 0.19) and Shannon indices (P = 0.056) (**Figure 2A**). Similar findings were observed in the analysis of all 44 study subjects (**Supplementary Figure S1A**). Furthermore, PCoA with PERMANOVA based on Bray-Curtis distance showed a trend toward significant differences in vaginal microbial communities between the two groups (R^2^ = 0.061, P = 0.069, **Figure 2B**). Upon expanding the sample size, a significant difference was confirmed (R^2^ = 0.065, P = 0.015, **Supplementary Figure S1B**).

The average relative abundance of dominant phyla, including *Firmicutes*, *Actinobacteria*, *Proteobacteria*, *Bacteroidetes*, and *Tenericutes*, was shown in **Figure 2C**. *Firmicutes* and *Actinobacteria* together accounted for over 90% of the microbial composition in both groups. At the genus level, the predominant taxa included *Lactobacillus*, *Gardnerella*, *Delftia*, *Atopobium*, *Methylosinus*, *Veillonella*, *Streptococcus*, *Acinetobacter*, *Methyloversatilis*, and *Aerococcus* (**Figure 2D**). Similar microbial distributions were observed across all 44 study subjects (**Supplementary Figures S1C, D**). LEfSe analysis revealed significant differences at the genus level between the two groups, with *Delftia*, *Burkholderia*, *Atopobium*, *Streptococcus*, *Stenotrophomonas* and *Pseudomonas* significantly enriched in the VVC group, while *Methyloversatilis*, *Methylosinus*, and *Lactobacillus* significantly decreased compared to the HC group (**Figure 2E**). Expanding sample size confirmed a significant increase in *Delftia*, *Burkholderia* and *Stenotrophomonas*, along with a significant decrease in *Methylosinus* and *Lactobacillus* were observed in the VVC group compared to the HC group (**Supplementary Figure S1E**).

### Comparisons of vaginal microbiome after delivery between the VVC and HC groups

**Figure 3:**
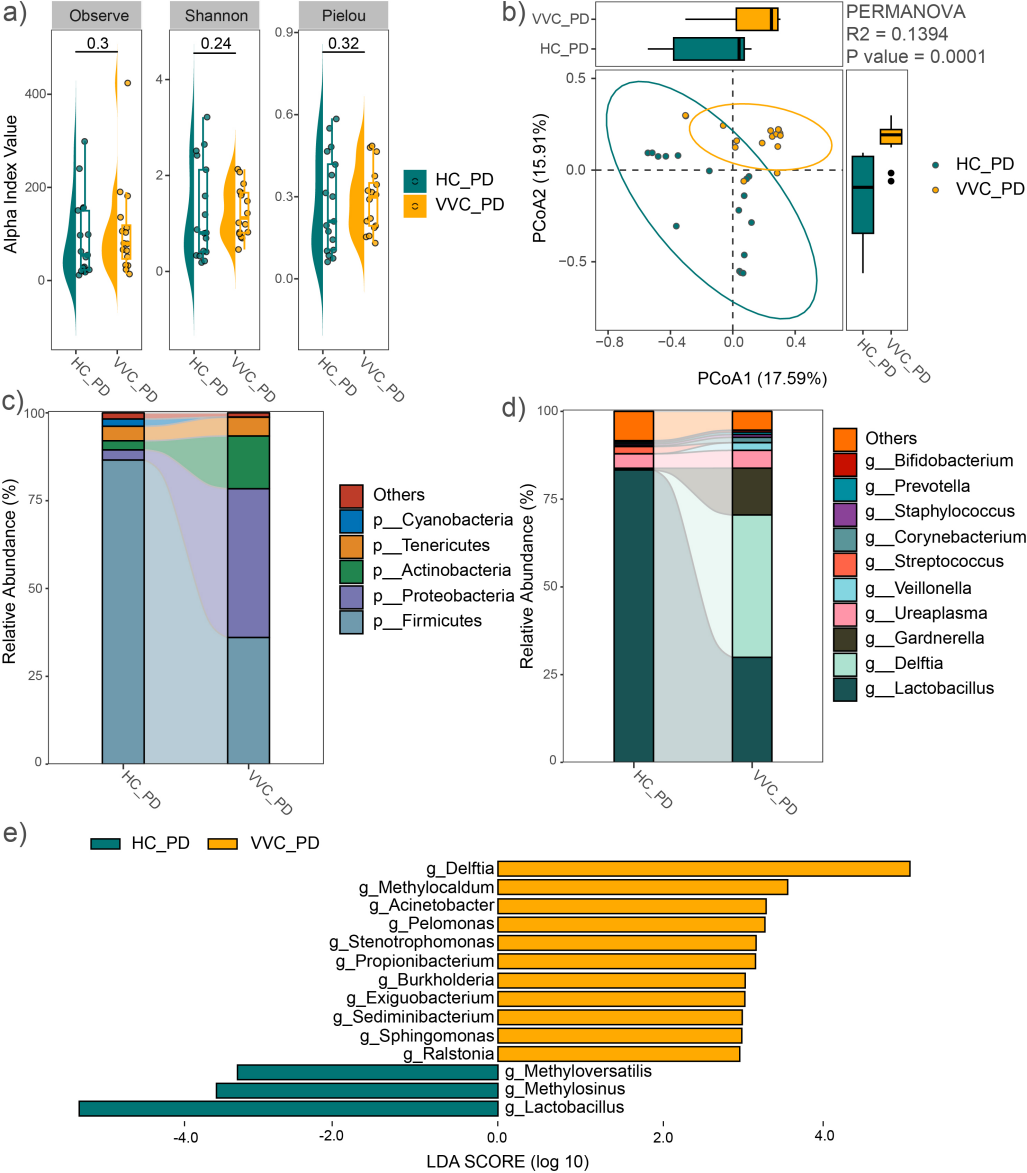
Comparison of vaginal microbial community after delivery between the VVC and HC groups. **(A)** Comparisons of alpha diversity indices. **(B)** PCoA plot illustrating the differences in microbial communities between the two groups. **(C)** Relative abundances of the dominant phyla. **(D)** Relative abundances of the predominant genus. **(E)** Significant different genus between the two groups.

After delivery, the mean values of the observed feature number, Pielou and Shannon indices showed no significant differences between the VVC and HC groups (**Figure 3A**). Consistent findings were observed in the analysis of all 44 study subjects (**Supplementary Figure S2A**). However, PCoA with PERMANOVA based on Bray-Curtis distance revealed significant differences in the vaginal microbial communities between the two groups (R^2^ = 0.1394, P = 0.0001, **Figure 3B**). When the sample size was expanded, this significant difference was further confirmed (**Supplementary Figure S2B**).

The average relative abundance of dominant phyla was shown in **Figure 3C**, similar to the vaginal microbiome before delivery, including *Firmicutes*, *Proteobacteria*, *Actinobacteria*, *Tenericutes*, and *Bacteroidetes*. The predominant genera included *Lactobacillus*, *Delftia*, *Gardnerella*, *Ureaplasma*, *Veillonella*, *Streptococcus*, *Corynebacterium*, *Staphylococcus*, *Prevotella*, *Enterococcus*, and *Bifidobacterium* (**Figure 3D**). These findings were confirmed across all study subjects (**Supplementary Figures S2C, D**). LEfSe analysis revealed significant genus-level differences between the two groups, with *Delftia*, *Methylocaldum*, *Acinetobacter*, *Pelomonas*, *Stenotrophomonas*, *Propionibacterium*, *Burkholderia*, *Exiguobacterium*, *Sediminibacterium*, *Sphingomonas*, and *Ralstonia* significantly enriched, while *Methyloversatilis*, *Methylosinus*, and *Lactobacillus* significantly decreased in the VVC group compared to the HC group (**Figure 3E**). These differences in genus were also confirmed with the expanded sample size (**Supplementary Figure S2E**).

Consistently, both before delivery and after delivery, *Delftia*, *Burkholderia*, and *Stenotrophomonas* were significantly enriched in the VVC group. All these genera belong to the phylum *Proteobacteria*. This was reflected in the differences at the phylum level, with *Proteobacteria* significantly enriched in the VVC group at both BD (LDA = 4.61, P < 0.001) and PD (LDA = 5.33, P < 0.001) phases.

### Comparisons of neonatal meconium microbiome between the VVC and HC groups

**Figure 4:**
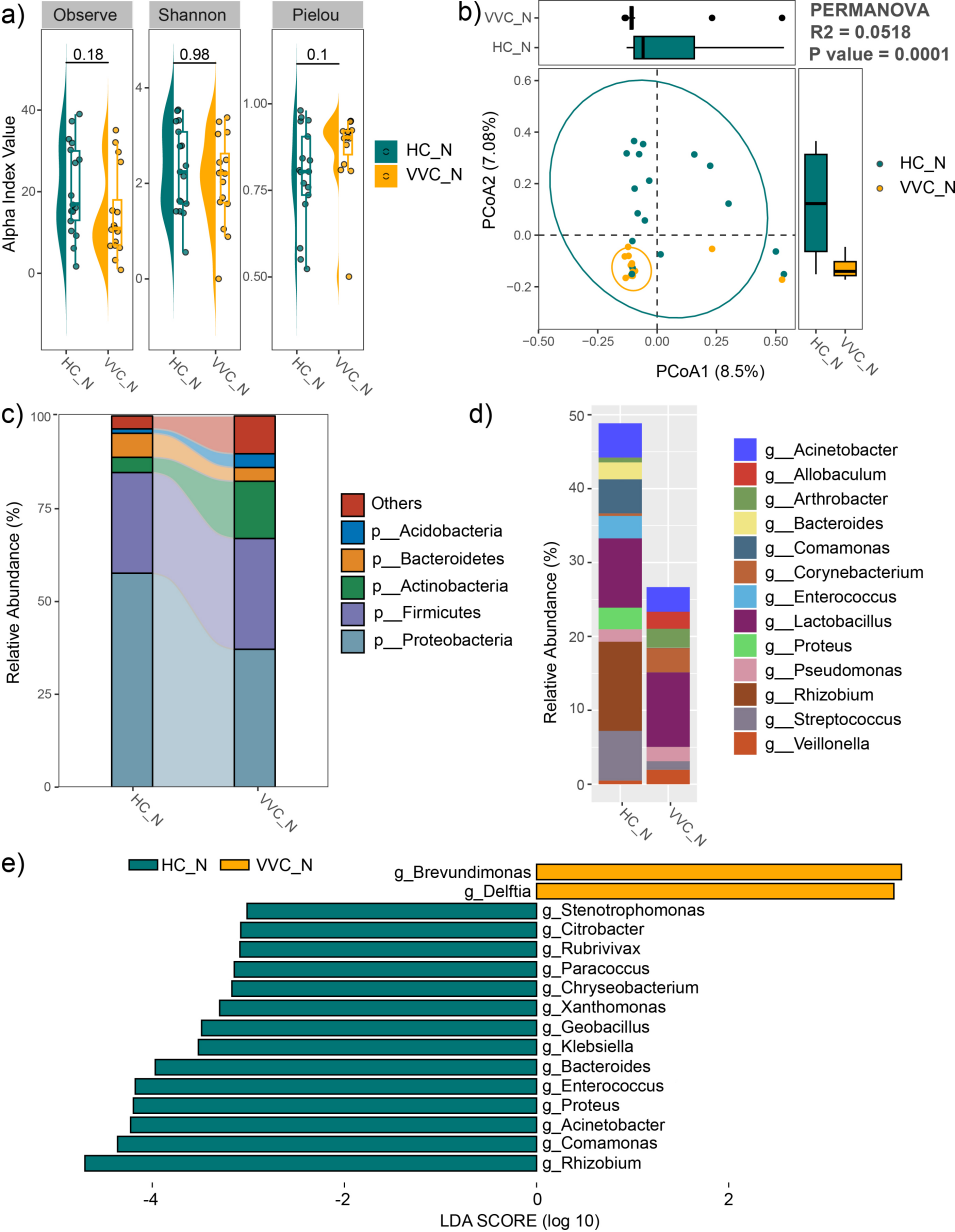
Comparison of neonatal meconium microbiome between the VVC and HC groups. **(A)** Comparisons of alpha diversity indices. **(B)** PCoA plot illustrating the differences in microbial communities between the two groups. **(C)** Relative abundances of the dominant phyla. **(D)** Relative abundances of the predominant genus. **(E)** Significant different genus between the two groups.

For the neonatal meconium microbiome, the mean values of the observed feature number, Pielou and Shannon indices showed no differences between the VVC and HC groups (**Figure 4A**). However, PCoA with PERMANOVA revealed significant differences in the neonatal meconium microbial communities between the two groups (R^2^ = 0.0518, P = 0.0001). The VVC group exhibited more closely clustered microbial communities, indicating a more homogeneous community structure among the neonates in the VVC group (**Figure 4B**).

The average relative abundance of dominant phyla was shown in **Figure 4C**, including *Proteobacteria*, *Firmicutes*, *Actinobacteria*, *Bacteroidetes*, and *Acidobacteria*. The predominant genus included *Veillonella*, *Streptococcus*, *Rhizobium*, *Proteus*, *Lactobacillus*, *Enterococcus*, *Corynebacterium*, *Comamonas*, *Bacteroides*, *Arthrobacter*, *Allobaculum* and *Acinetobacter* (**Figure 4D**). LEfSe analysis revealed significant differences at the genus level between the two groups, with *Brevundimonas*, and *Delftia* significantly enriched, while *Stenotrophomonas*, *Citrobacter*, *Rubrivivax*, *Paracoccus*, *Chryseobacterium*, *Xanthomonas*, *Geobacillus*, *Klebsiella*, *Bacteroides*, *Enterococcus*, *Proteus*, *Acinetobacter*, *Comamonas* and *Rhizobium* significantly decreased in the VVC group compared to the HC group (**Figure 4E**).

### Lactobacillus species’ differences between the VVC and HC groups

It is well-established that the female genital tract is predominantly inhabited by *Lactobacillus* species, which protect against infections through the production of L-lactic acid and H_2_O_2_. Pathological changes in their profile may render the vagina susceptible to infections. In this study, we identified various *Lactobacillus* species, including *L. iners*, *L. helveticus*, *L. reuteri*, *L. zeae*, *L. coleohominis*, *L. mucosae*, *L. manihotivorans*, *L. salivarius* and *L. acidipiscis* (**Supplementary Figure S3**). Among these, *L. iners* and *L. helveticus* were the most abundant species in both vaginal and meconium samples (**Supplementary Figure S3**). Differences in these *Lactobacillus* species were further investigated using LEfSe analysis. At the BD phase, no significant differences in the relative abundance of these *Lactobacillus* species were observed. However, the overall abundance of the genus *Lactobacillus* was significantly decreased in the VVC group compared to the HC group (LDA = 5.24, P = 0.029), a result confirmed across all 44 study subjects (LDA = 5.14, P = 0.027). A similar pattern was observed at the PD phase, with a significant decrease in the genus *Lactobacillus* in the VVC group (LDA = 5.44, P < 0.01), particularly in species *L. helveticus* (LDA = 5.20, P = 0.014). Only the significant difference in genus *Lactobacillus* was confirmed with the expanded sample size (LDA = 5.32, P = 0.001). In contrast, neonatal meconium samples showed no significant differences of the genus *Lactobacillus*, though *L. unclassified* (LDA = 2.67, P = 0.021), *L. salivarius* (LDA = 2.85, P = 0.042), *L. helveticus* (LDA = 4.47, P = 0.023) were significantly more abundant in the HC group.

### Comparisons of vaginal microbiome between the pre- and post- delivery phases

**Figure 5:**
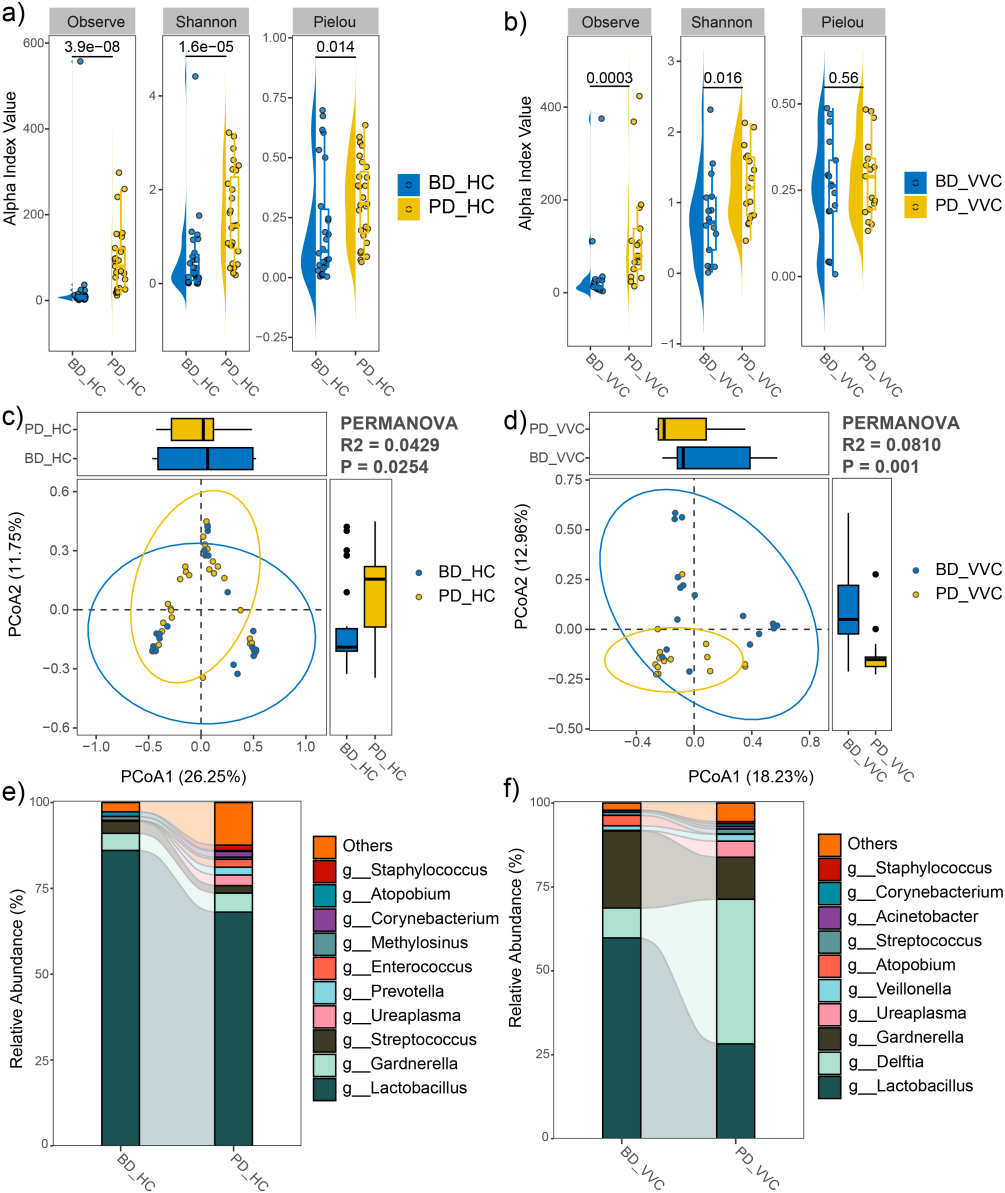
Comparison of vaginal microbial community between pre- and post- delivery phases in both the HC and VVC groups. **(A)** Alpha diversity indices between two phases in the HC group. **(B)** Alpha diversity indices between two phases in the VVC group. **(C)** PCoA plot illustrating the differences in microbial communities between the two phases in the HC group. **(D)** PCoA plot illustrating the differences in microbial communities between the two phases in the VVC group. **(E)** Relative abundances of the predominant genus in the HC group, a decrease of *Lactobacillus* was observed. **(F)** Relative abundances of the predominant genus in the VVC group, a decrease of *Lactobacillus* was observed.

In both the VVC and HC groups, the observed feature number and Shannon index significantly increased in the PD phase compared to the BD phase (**Figures 5A, B**), indicating an increase of microbial diversity after delivery. Moreover, PCoA with PERMANOVA based on Bray-Curtis distance revealed significant differences in the vaginal microbial communities between pre- and post- delivery (R^2^ = 0.0429, P = 0.0254 in the HC group, **Figure 5C**; R^2^ = 0.0810, P = 0.001 in the VVC group, **Figure 5D**). Additionally, after delivery, the relative abundance of *Lactobacillus* significantly decreased in both the HC and VVC groups (LDA = 4.98, P < 0.001 in the HC group, **Figure 5E**; LDA = 5.18, P = 0.026 in the VVC group; **Figure 5F**).

## Discussion

This study aimed to characterize the vaginal microbial profile in pregnant women affected by vulvovaginal candidiasis and its effect on their offspring. The results revealed significant differences in the vaginal microbiome associated with VVC, as evidenced by PCoA based on the Bray-Curtis dissimilarity index at both pre- and post-delivery phases. Additionally, this study showed that the vaginal microbiome of most women in HC group was dominated by *Lactobacillus*, consistent with the typical vaginal microbiota composition in healthy women as previously reported (DiGiulio et al., 2015; MacIntyre et al., 2015; Zhang et al., 2022a). The significant decrease in the relative abundance of *Lactobacillus* observed in the VVC group is consistent with previous studies (Ceccarani et al., 2019). Moreover, the meconium microbiome of the offspring of women with VVC also presented significant differences compared to the healthy control group, with *L. salivarius* and *L. helveticus* significantly decreased. *Lactobacillus* species help maintain an acidic environment (low pH) in the vagina, which is essential for preventing the overgrowth of harmful bacteria, yeast, and viruses (Wu et al., 2024). These bacteria produce substances such as lactic acid, hydrogen peroxide, and bacteriocins that inhibit the growth of pathogenic microorganisms (Pendharkar et al., 2023). *Lactobacillus* can also form biofilms that act as a physical barrier against pathogens, providing an additional layer of protection (Chee et al., 2020). *L. salivarius* and *L. helveticus* are types of lactic acid bacteria commonly used as probiotic for infants (Manzano et al., 2017; Chen et al., 2023). These *Lactobacillus* species aid digestion and nutrient absorption, enhance the production of secretory immunoglobulin A, which are essential for neonatal growth and development, and help reduce the risk of infections and inflammation (Gritz and Bhandari, 2015). Additionally, they support the maturation of the neonatal immune system. The significant decrease in these bacteria in both maternal vaginal and neonatal gut microbiome may be closely related to their health status.

Accompanying with the decrease of *Lactobacillus* in the vaginal microbiome of the VVC group was a significant increase in genera such as *Delftia* and *Burkholderia* from the phylum Proteobacteria. *Delftia* species, which have been isolated from vaginal discharge, are found related to cervical intra-epithelial neoplasia and unexplained recurrent pregnancy loss (Wu et al., 2020; Mori et al., 2023). Some strains of *Delftia* carry multiple antibiotic resistance genes, complicating treatment if infections occur (Zhang et al., 2024). An increase in *Burkholderia* is associated with negative reproductive outcomes, such as recurrent implantation failure in assisted reproduction technology procedures (Chen et al., 2024). Interestingly, a significant increase in *Delftia* was observed in the meconium microbiome of the offspring of women with VVC. Some species of *Delftia* are pathogenic to infants and have been associated with respiratory infection (Ranc et al., 2018; Agarwal et al., 2023). Unfortunately, this study was unable to identify the specific *Delftia* species based on 16S sequencing data, which limited further analysis. Collectively, their increase may be harmful to maternal and neonatal health.

An interesting finding in this study was the significant alterations of vaginal microbiome observed between the pre- and post- delivery phases in both the HC and VVC groups, with a notable decrease in *Lactobacillus* abundance after delivery. This is consistent with previous reports by Li et al (Li et al., 2022, 2023), which suggest associations with povidone-iodine use during delivery. Our results provide additional evidence supporting these findings.

Furthermore, the significant decrease in *Lactobacillus* species and the concomitant increase in *Delftia* species observed in the VVC group were consistent across both the maternal vaginal microbiome and neonatal meconium microbiome. This parallel in microbial shifts between mothers and their neonates suggests a potential intergenerational transmission associated with VVC. However, to validate these findings, it is essential to enlarge the sample size and recruit participants from multiple hospitals to account for potential confounding factors such as diet and demographic distances. Additionally, conducting metagenomic sequencing would provide deeper insights into the observed alterations in the maternal vaginal and neonatal meconium microbiome with VVC. Collecting comprehensive health data for both mothers and their offspring in future studies will also be crucial. These steps would strengthen the robustness and applicability of the study’s findings, paving the way for more comprehensive and insightful research in this critical area.

In conclusion, a notable distinction was observed in the maternal vaginal microbiome and neonatal meconium microbiome between VVC and healthy control groups. The persistence of these differences and their potential impact on growth and development will be the focus of forthcoming studies. This ongoing investigation will shed further light on the intricate relationship between vaginal microbial composition in pregnant women with VVC.

## Data Availability

The datasets presented in this study can be found in online repositories. The names of the repository/repositories and accession number(s) can be found below: https://db.cngb.org/search/project/CNP0006080.
