# Supplementary material for: Alterations of the paired maternal vaginal microbiome and neonatal meconium microbiome in vulvovaginal candidiasis positive pregnant women
Source: Front Cell Infect Microbiol. 2024 Dec 13;14:1480200. doi: 10.3389/fcimb.2024.1480200 (PMC11673766; doi:10.3389/fcimb.2024.1480200)
Supplement: Supplementary file 1 [file DataSheet1.docx]

**Supplementary Figures for**

**Alterations of the paired maternal vaginal microbiome and neonatal meconium microbiome in vulvovaginal candidiasis positive pregnant women**

Hongqin Zhang^1#^, Hongping Li^2#^, Ruolin Zhang^1^, Lingxia Ji^1^, Jun Chen^1^, Chuan Nie^3*^, Weimin Huang^2*^

^1^ Neonatology Department, Shenzhen Nanshan Maternity and Child Healthcare Hospital, Shenzhen, China

^2^ Neonatology Department, Affiliated Shenzhen Children’s Hospital of Shantou University Medical College, Shenzhen, China

^3^ Neonatology Department, Guangdong Women and Children Hospital, Guangzhou, China

^#^ Contributed equally to this work

^*^ Corresponding author, Weimin Huang, Email: hwmnet@21cn.com, Address: No. 7019, Yitian Road, Futian District, Shenzhen, China

Chuan Nie, Email: chuannie@sina.com, Address: No. 13, Guangyuan West Road, Yuexiu District, Guangzhou, China

**
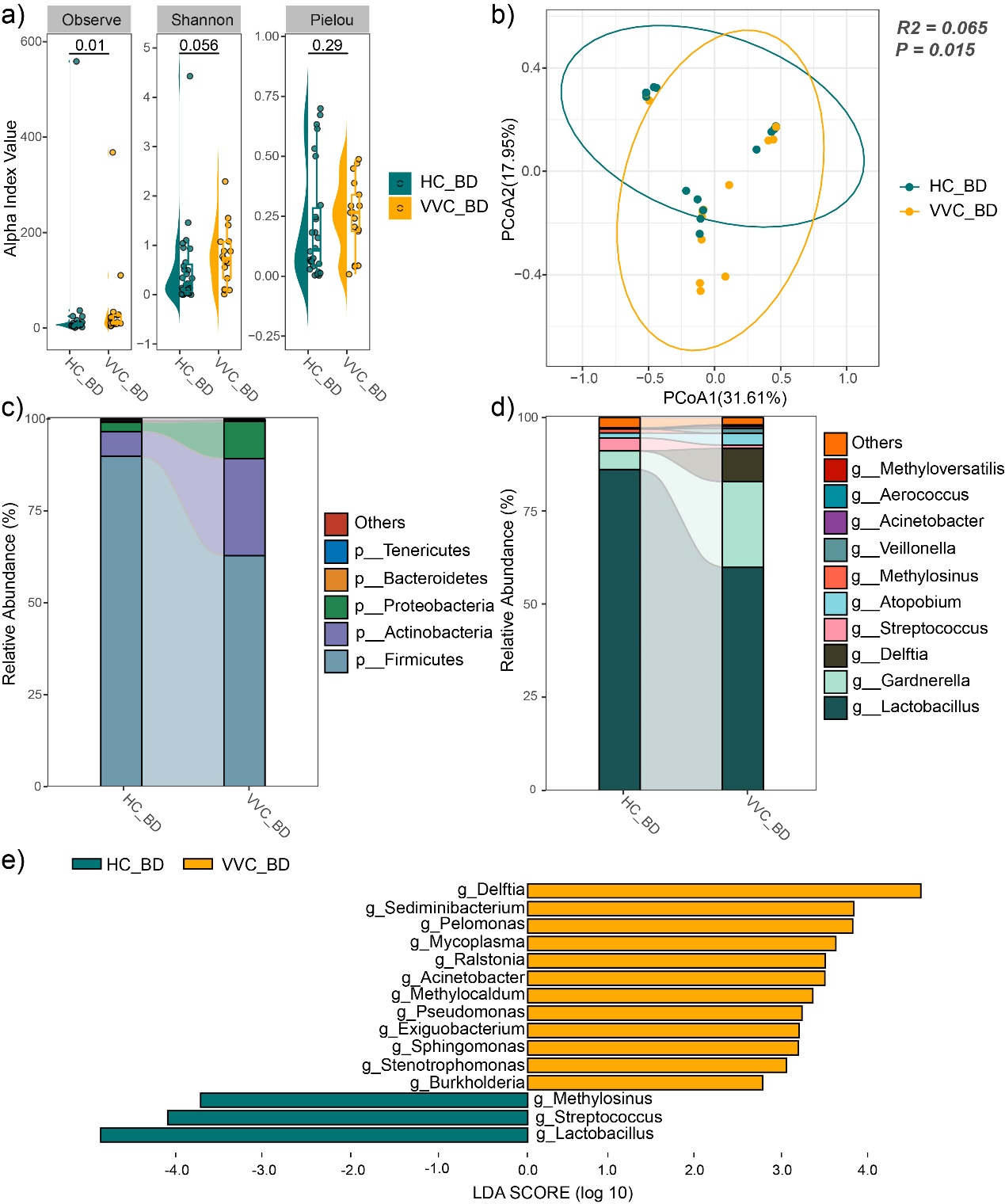
**

Figure S1. Comparison of vaginal microbial community before delivery between the VVC and HC groups across all study subjects. a) Comparisons of alpha diversity indices. b) PCoA plot illustrating the differences in microbial communities between the two groups. c) Relative abundances of the dominant phyla. d) Relative abundances of the predominant genus. e) Significant different genus between the two groups.


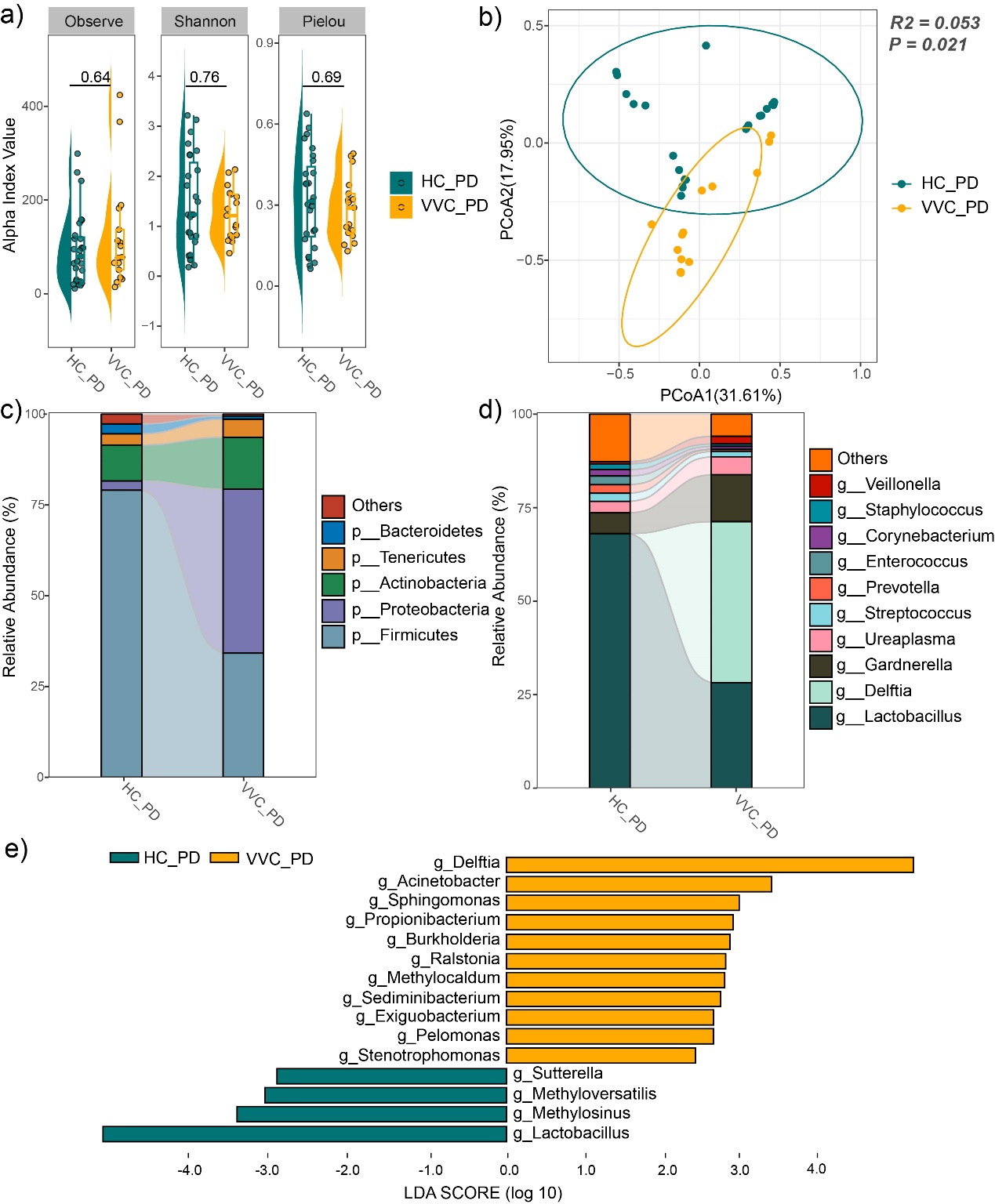


Figure S2. Comparison of vaginal microbial community after delivery between the VVC and HC groups across all study subjects. a) Comparisons of alpha diversity indices. b) PCoA plot illustrating the differences in microbial communities between the two groups. c) Relative abundances of the dominant phyla. d) Relative abundances of the predominant genus. e) Significant different genus between the two groups.


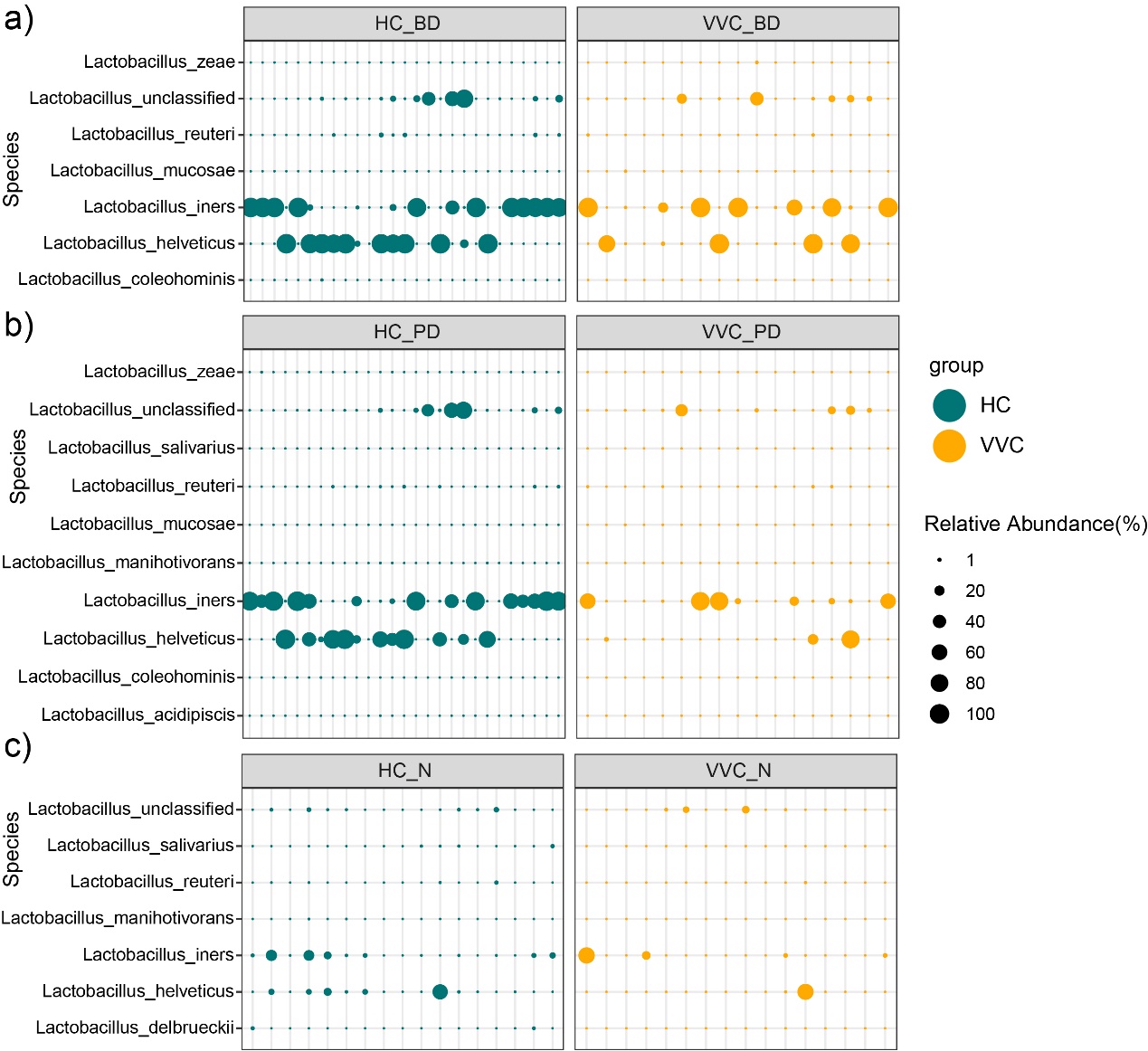


Figure S3. Comparison of Lactobacillus species between the VVC and HC groups in vaginal and meconium samples, respectively. a) Visualization of Lactobacillus species in each vaginal sample at the BD phase. b) Visualization of Lactobacillus species in each vaginal sample at the PD phase. c) Visualization of Lactobacillus species in each meconium sample.
